# Supplementary material for: Restoration of susceptibility to amikacin by 8-hydroxyquinoline analogs complexed to zinc
Source: PLoS One. 2019 May 29;14(5):e0217602. doi: 10.1371/journal.pone.0217602 (PMC6541283; doi:10.1371/journal.pone.0217602)
Supplement: S1 Fig — (PDF) [file pone.0217602.s001.pdf]

Supporting Information

Restoration of Susceptibility to Amikacin by 8-Hydroxyquinoline Analogs Complexed to Zinc

Jesus Magallón, Kevin Chiem, Tung Tran, Kimberly Phan, María S. Ramirez, Verónica Jimenez, and Marcelo E. Tolmasky*

Center for Applied Biotechnology Studies, Department of Biological Science, College of Natural Sciences and Mathematics, California State University Fullerton, Fullerton, CA 92834-6850, United States

S1 Fig, A

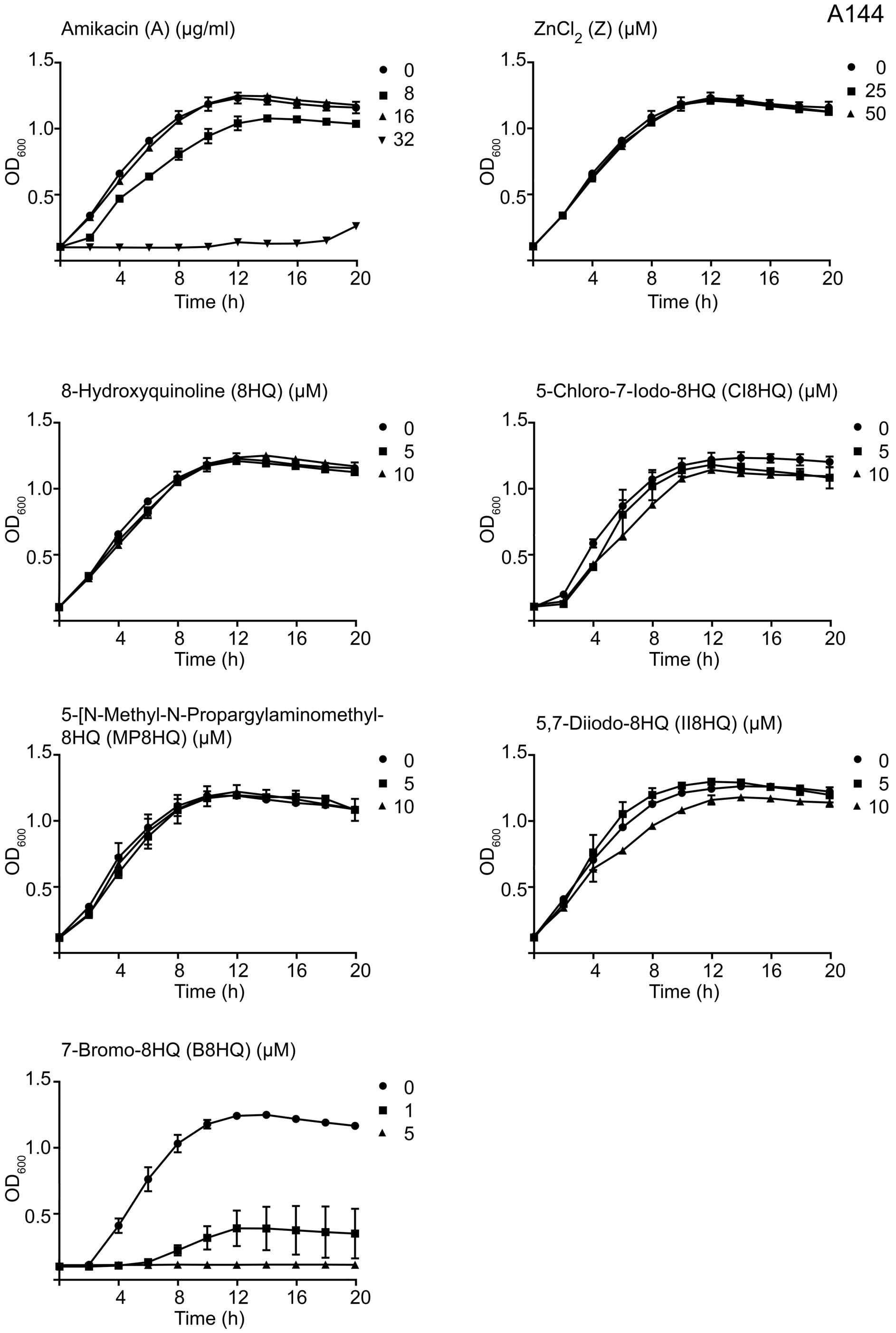
A144
Amikacin (A) (µg/ml)
ZnCl2 (Z) (µM)
8-Hydroxyquinoline (8HQ) (µM)
5-Chloro-7-Iodo-8HQ (CI8HQ) (µM)
5-[N-Methyl-N-Propargylaminomethyl-8HQ (MP8HQ) (µM)
5,7-Diiodo-8HQ (II8HQ) (µM)
7-Bromo-8HQ (B8HQ) (µM)
OD600
Time (h)
0
8
16
32
25
50
5
10
1

S1 Fig, B

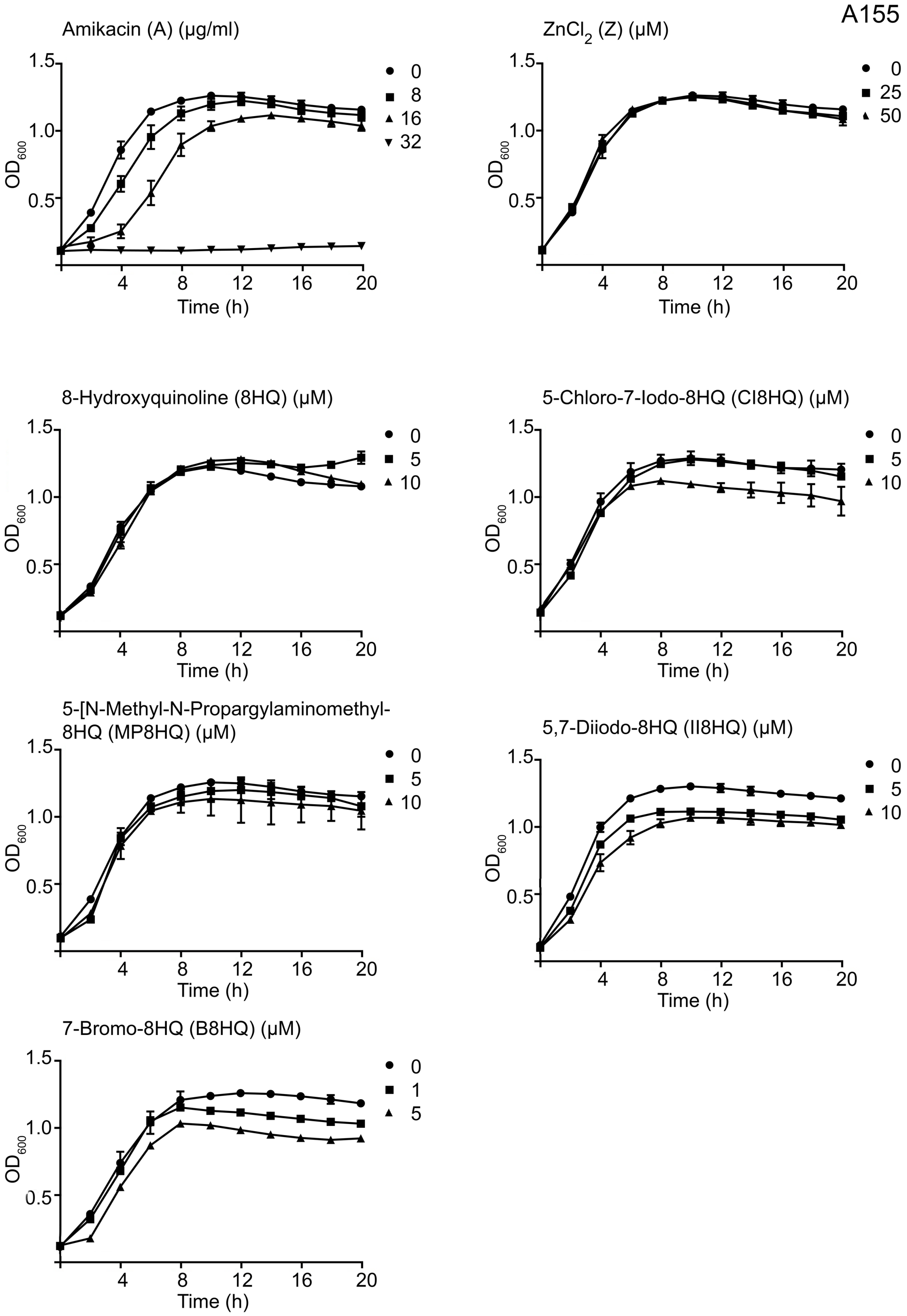
A155
Amikacin (A) (µg/ml)
0
8
16
32
ZnCl2 (Z) (µM)
0
25
50
8-Hydroxyquinoline (8HQ) (µM)
0
5
10
5-Chloro-7-Iodo-8HQ (CI8HQ) (µM)
0
5
10
5-[N-Methyl-N-Propargylaminomethyl-8HQ (MP8HQ) (µM)
0
5
10
5,7-Diiodo-8HQ (II8HQ) (µM)
0
5
10
7-Bromo-8HQ (B8HQ) (µM)
0
1
5
OD600
Time (h)
0.5
1.0
1.5
4
8
12
16
20

S1 Fig, C

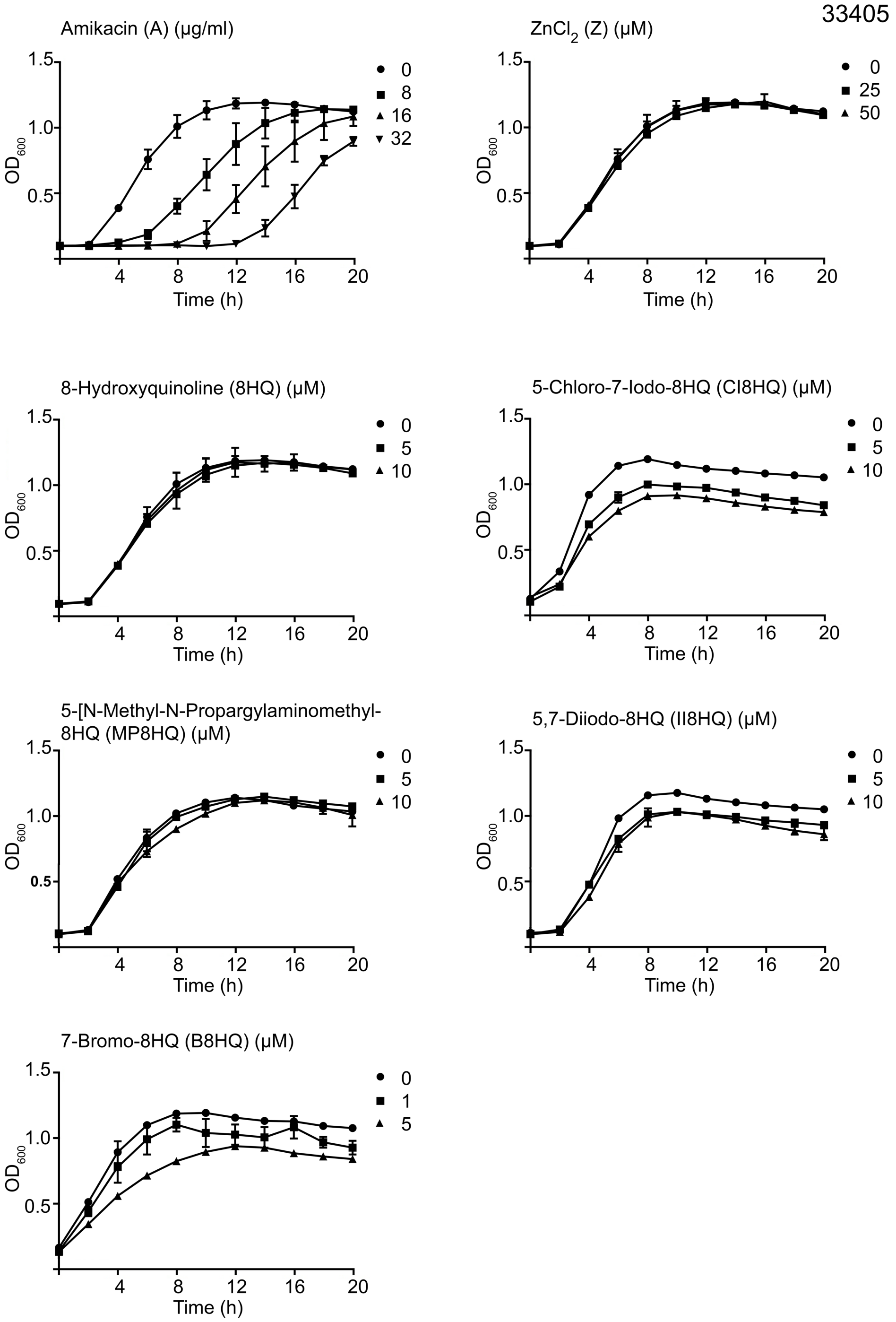

Fig S1. Effect of addition of different reagents on growth of *A. baumannii* strains. *A. baumannii* A144 (A), A155 (B) or 33405 (C) were cultured in 100 μl Mueller-Hinton broth in microtiter plates at 37°C, with the additions indicated in the figure and the OD_{600} values were periodically determined.
