# Supplementary material for: Restoration of susceptibility to amikacin by 8-hydroxyquinoline analogs complexed to zinc
Source: PLoS One. 2019 May 29;14(5):e0217602. doi: 10.1371/journal.pone.0217602 (PMC6541283; doi:10.1371/journal.pone.0217602)
Supplement: S2 Fig — (PDF) [file pone.0217602.s002.pdf]

Supporting Information

Restoration of Susceptibility to Amikacin by 8-Hydroxyquinoline Analogs Complexed to Zinc

Jesus Magallón, Kevin Chiem, Tung Tran, Kimberly Phan, María S. Ramirez, Verónica Jimenez, and Marcelo E. Tolmasky*

Center for Applied Biotechnology Studies, Department of Biological Science, College of Natural Sciences and Mathematics, California State University Fullerton, Fullerton, CA 92834-6850, United States

S2 Fig

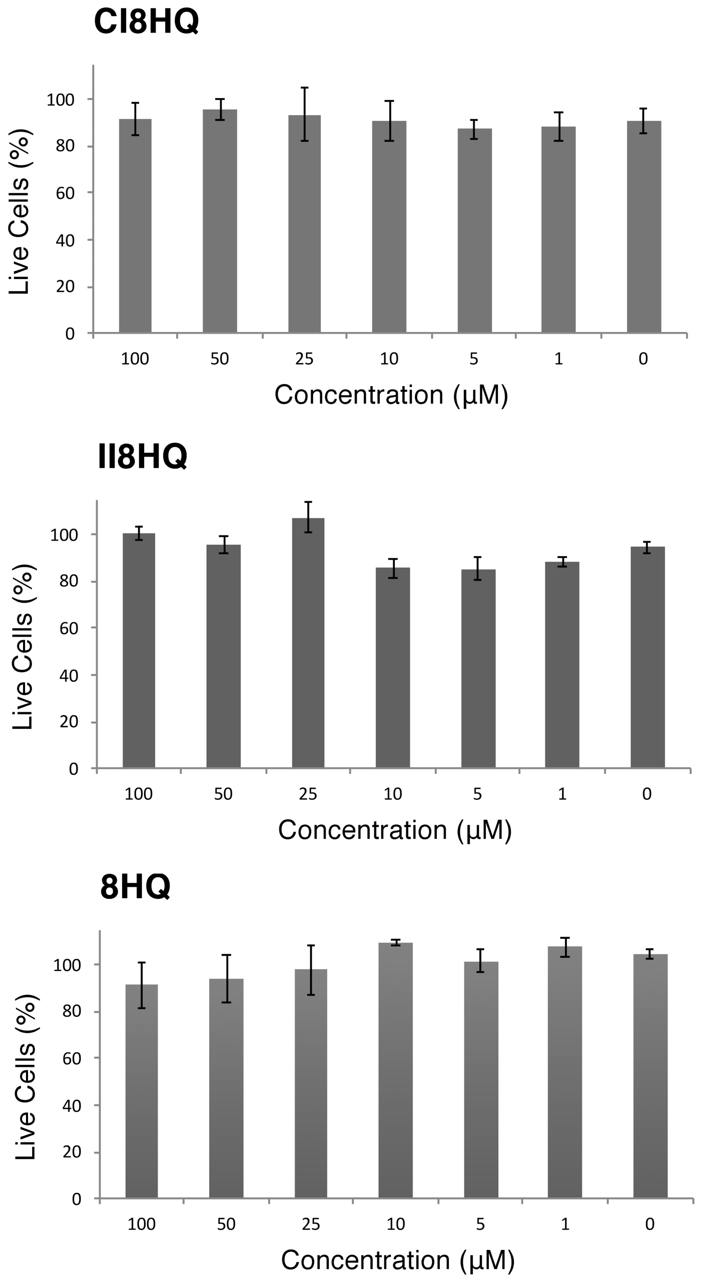

Fig S2. Cytotoxicity tests. Cytotoxicity on HEK293 cells treated with the indicated concentrations of the different compounds for 24 h was assayed using a LIVE/DEAD kit. The percentage of dead cells was calculated relative to the cells treated with DMSO. Cells incubated with 0.1% Triton X-100 for 10 min were used as a control for maximum toxicity. Experiments were conducted in triplicate and the values are mean ± SD (n=5).
